# Mathematical Model of the Effect of Interstitial Fluid Pressure on Angiogenic Behavior in Solid Tumors

**DOI:** 10.1155/2011/843765

**Published:** 2011-09-07

**Authors:** Colin Phipps, Mohammad Kohandel

**Affiliations:** ^1^Department of Applied Mathematics, University of Waterloo, Waterloo, ON, Canada N2L 3G1; ^2^Center for Mathematical Medicine, Fields Institute for Research in Mathematical Sciences, Toronto, ON, Canada M5T 3J1

## Abstract

We present a mathematical model for the concentrations of proangiogenic and antiangiogenic growth factors, and their resulting balance/imbalance, in host and tumor tissue. In addition to production, diffusion, and degradation of these angiogenic growth factors (AGFs), we include interstitial convection to study the locally destabilizing effects of interstitial fluid pressure (IFP) on the activity of these factors. The molecular sizes of representative AGFs and the outward flow of interstitial fluid in tumors suggest that convection is a significant mode of transport for these molecules. The results of our modeling approach suggest that changes in the physiological parameters that determine interstitial fluid pressure have as profound an impact on tumor angiogenesis as those parameters controlling production, diffusion, and degradation of AGFs. This model has predictive potential for determining the angiogenic behavior of solid tumors and the effects of cytotoxic and antiangiogenic therapies on tumor angiogenesis.

## 1. Introduction

The process of angiogenesis, the development of new blood vessels from preexisting vasculature, is governed by the net balance between proangiogenic and antiangiogenic growth factors [1]. Multiple regulatory factors are involved in this process, including the vascular endothelial growth factor (VEGF) family and its receptors, the fibroblast growth factor (FGF) family, angiostatin, and endostatin [1]. During tumor progression, this delicate balance is skewed in favor of angiogenesis, resulting in an abnormal tumor vasculature and microenvironment [2, 3].

The tumor vascular network is spatially and temporally heterogeneous, and hypoxia, acidosis, and elevated interstitial fluid pressure (IFP) are characteristic features of solid tumors. While it is known that all three of these traits play critical roles in the activity and upregulation of angiogenic growth factors (AGFs), the relationships between these features and tumor angiogenesis are complex and not fully understood. The most prominent and widely studied AGF is VEGF, a potent proangiogenic agent that is independently upregulated by both hypoxia and acidosis [4]. While angiogenesis is commonly triggered as a result of hypoxia, it is also the case that acidic pH induces several angiogenic molecules such as basic FGF [5] and nitric oxide [6]. Although the effects of hypoxia and acidosis on angiogenic factors have been investigated experimentally, the effects of elevated IFP are less clearly understood. Here, we seek to explore the effect of IFP gradients on proangiogenic and antiangiogenic factor concentrations, with a focus on the angiogenic tendency induced by their imbalance.

Two compounding factors that contribute to elevated IFP in solid tumors are the increased permeability of blood vessels and the absence of functional lymphatics [3, 7]. It has been hypothesized that the normalization of tumor vessels, by the application of antiangiogenic therapies such as antibodies inhibiting VEGF or blocking VEGFR-2, would lead to lowered tumor IFP [2, 8]. Baxter and Jain [9] developed a mathematical model to study the transport of fluid and macromolecules in tumors. Recently, Jain et al. [10] revisited their model to investigate the effect of vascular normalization by antiangiogenic therapy on IFP, as well as to determine the parameters that could lead to a reduction of IFP.

In other work, Ramanujan et al. [11] used a mathematical framework to study the local imbalance of pro- and antiangiogenic factors. Their model contained production, diffusion, and degradation of these factors, and was used to explain focal necrosis and dormancy in tumors. Here, we generalize their model and include the effect of interstitial convection on proangiogenic and antiangiogenic factor concentrations, which is supported by the fact that convection can contribute significantly to the transport of molecules of the typical size of AGFs (see Table 1). It has also been noted that convection plays an important role, not only in determining local concentrations, but also in the functionality and activity of AGFs, particularly VEGF [12]. This effect is added via a convection term in the equations that govern the concentrations of AGFs (see following section). The mathematical model is then used to study the changes in a tumor's angiogenic behavior as a result of altering tissue or blood vessel properties (e.g., hydraulic conductivity of tissue, hydraulic permeability of vessels).

## 2. Materials and Methods

### 2.1. Angiogenic Growth Factor Model

Following Hahnfeldt et al. [18], and Ramanujan et al. [11], we first assume that angiogenic growth factors can be considered to initiate either proangiogenic (stimulatory) or antiangiogenic (inhibitory) activity. Although some of these factors may behave in either a proangiogenic or antiangiogenic manner depending on the state of the system, we consider this effect to be negligible. While different cellular mechanisms and signaling cascades activated by specific (and possibly multiple) factors have greater effects on the level of angiogenic activity than others, we simply consider the pro- and antiangiogenic factor concentrations to be representative of the angiogenic effect enabled by these two categories. The proangiogenic (*p*) and antiangiogenic (*a*) factor concentrations will be denoted by *f*_*p*_ and *f*_*a*_ (*μ*g/mm^3^), respectively. These factors are assumed to diffuse with constant diffusion coefficients *D*_*j*_ (mm^2^/s), *j* = *p*, *a*, to degrade under first-order kinetics with constant deactivation rates *k*_*j*_ (s^−1^) and to be produced independently with constant production rates *g*_*j*_ (*μ*g/mm^3^/s). Since the aforementioned diffusion, deactivation, and production parameters are used to describe entire families of factors with varying molecular weights and kinetic rates, they are assumed to be representative of their respective angiogenic categories. However, each of these parameters can differ in host and tumor tissue, both of which are assumed to be homogeneous and isotropic, so that when appropriate, we will distinguish between these values with superscripts for host (*h*) and tumor (*t*) tissues. As indicated earlier, we also include the convection of these factors since interstitial fluid velocity (IFV) in the tumor plays a role in determining the concentration of macromolecules including AGFs in the tumor. Assuming the velocity of these molecules is equal to the interstitial fluid velocity *u* (mm/s) [19], we arrive at the equation

(1) $$\frac{\partial f_{j}}{\partial t}=D_{j}\nabla^{2}f_{j}-k_{j}f_{j}+g_{j}-\nabla \cdot \left(uf_{j}\right),\;j=p,a,$$

where the IFV is given by Darcy's law: *u* = −*K*∇*p*, where *K* (mm^2^/s/mm Hg) is the hydraulic conductivity of the interstitium and *p* (mm Hg) is the IFP [20, 21]. The equations governing IFP are derived below and presented in (5). By adding convection to this equation, we can study larger tumors since the model was previously restricted to those where convection was not a factor (usually those tumors with radii less than 2 mm). It is worthwhile reemphasizing that interstitial convection also plays a vital role in the activity of AGFs and the process of tumor angiogenesis [12]. Convective effects have been incorporated in previous models of drug distribution and other macromolecules in tumors [9, 20], and thus it seems natural to include it in this case. The parameters for the diffusion, production, and degradation of AGFs inside the host and tumor tissues are those assumed based on the conditions stated in Ramanujan et al. [11] and these are presented in Table 2.

To facilitate solving (1), we assume that the tumor is a sphere of radius *R* (mm), and since the dynamics of growth factor distribution occur on a much faster time scale than tumor growth, we consider the system in steady state. This results in the equation

(2) $$\frac{D_{j}}{r^{2}}\frac{d}{dr}\left(r^{2}\frac{df_{j}}{dr}\right)-k_{j}f_{j}+g_{j}+\frac{K}{r^{2}}\frac{d}{dr}\left(r^{2}\frac{dp}{dr}f_{j}\right)=0.$$

Equation (2) can be nondimensionalized by setting $\tilde{r}=r/R$ and ${\tilde{f}}_{j}=f_{j}/f_{j}^{s}$ where *f*_*j*_^*s*^ = *g*_*j*_^*h*^/*k*_*j*_^*h*^ is the steady-state AGF concentration in host tissue. Dividing by *D*_*j*_ eliminates the diffusion coefficient and gives the nondimensional parameters ${\tilde{k}}_{j}=k_{j}R^{2}/D_{j}$, ${\tilde{g}}_{j}=g_{j}R^{2}/(D_{j}f_{j}^{s})$ and, ${\tilde{K}}_{j}=Kp_{e}/D_{j}$ where *p*_*e*_ (mm Hg) is the effective pressure used to nondimensionalize the equation for pressure (the nondimensional pressure is defined to be $\tilde{p}=p/p_{e}$, refer to (6)). This nondimensionalization yields the equation

(3) $$\frac{1}{{\tilde{r}}^{2}}\frac{d}{d\tilde{r}}\left({\tilde{r}}^{2}\frac{d{\tilde{f}}_{j}}{d\tilde{r}}\right)-{\tilde{k}}_{j}{\tilde{f}}_{j}+{\tilde{g}}_{j}+\frac{{\tilde{K}}_{j}}{{\tilde{r}}^{2}}\frac{d}{d\tilde{r}}\left({\tilde{r}}^{2}\frac{d\tilde{p}}{d\tilde{r}}{\tilde{f}}_{j}\right)=0.$$

This step is essential to the model since the nondimensional quantities are used to define a measure of angiogenic activity below in (4).

In the absence of the convection term, that is, eliminating the last term in (3), an analytical solution can be obtained with appropriate boundary conditions [11]. However, in this work, we also consider the scenario where diffusion and convection could play a significant role in factor transport, and hence we rely on numerical integration schemes to solve (3).

### 2.2. Angiogenic Activity

While we are interested in the qualitative effects of IFP on AGF concentrations, we are not specifically interested in the quantitative concentrations of these two factor groups. The relationship between the proangiogenic and antiangiogenic forces is of greater importance, since the balance between these factors is the determinant of whether angiogenesis will be locally suppressed or initiated. Following Stoll et al. [22], we introduce a measure of angiogenic activity *a* defined by

(4) $$a=\{\begin{array}{c} \frac{{\tilde{f}}_{p}}{{\tilde{f}}_{a}}-1,\begin{array}{cc} & {\tilde{f}}_{p}\geq {\tilde{f}}_{a} \end{array},\\ 1-\frac{{\tilde{f}}_{a}}{{\tilde{f}}_{p}},\begin{array}{cc} & {\tilde{f}}_{p}<{\tilde{f}}_{a} \end{array}, \end{array}$$

where *a* > 0 corresponds to angiogenesis being initiated, *a* = 0 indicates a stable vasculature network, and *a* < 0 implies that no angiogenesis is taking place, and vessels could be regressing. While other forms of this function for *a* are viable, we choose this form due to its symmetry and the apparent inclusion of the balance between these nondimensionalized factor concentrations. 

A typical angiogenic activity scenario in a solid tumor maintains angiogenic repression at the tumor core where heightened levels of angiogenic inhibitors override the effect of elevated proangiogenic factor production. They also exhibit angiogenic stimulation near the tumor boundary where the angiogenic balance leans toward a proangiogenic tendency. This typically leads to the development of both an oxygen-deprived core consisting of hypoxic and necrotic cells along with a heavily vascularized and rapidly proliferating outer rim [23].

We use the angiogenic activity measure *a* to classify the model output into one of three cases: focal suppression, global suppression, and global angiogenesis [11]. The typical focally suppressive behavior described above is characterized by a transition from negative to positive values of *a* as we move from the core to the rim part of the tumor. Global suppression and angiogenesis are defined by *a* < 0 and *a* > 0, respectively, everywhere inside the tumor (i.e., for all *r* ∈ [0, *R*]).

### 2.3. Interstitial Fluid Pressure

We assume that, as previously mentioned, Darcy's law gives the relationship between interstitial fluid velocity *u* and interstitial fluid pressure *p* in an isotropic and homogeneous tissue: *u* = −*K*(*dp*/*dr*), where *K* is the hydraulic conductivity of the interstitium (mm^2^/s/mm Hg). The continuity equation for steady-state incompressible flow is ∇·*u* = *ϕ*(*r*) where *ϕ*(*r*) is the fluid source term (s^−1^). We assume a continuous distributed source throughout the tumor given by Starling's law: *ϕ*(*r*) = (*L*_*p*_ *S*/*V*)(*p*_*v*_ − *p* − *σ*(*π*_*v*_ − *π*_*i*_)). Here, the parameters are the hydraulic permeability of the microvascular wall *L*_*p*_ (mm/s/mm Hg), the surface area of vessel wall per unit volume of tumor *S*/*V* (mm^2^/mm^3^), the vascular pressure *p*_*v*_ (mm Hg), and the average osmotic reflection coefficient for plasma proteins *σ*. The plasma and interstitial osmotic pressures are denoted by *π*_*v*_ and *π*_*i*_ (mm Hg), respectively. Assuming *K* is also constant, we substitute Darcy's law and Starling's law into the continuity equation to obtain the model equation for IFP in a solid tumor [9]

(5) $$\nabla^{2}p=-\frac{L_{p}}{K}\frac{S}{V}\left(p_{v}-\sigma \left(\pi_{v}-\pi_{i}\right)-p\right).$$

Equation (5) can be nondimensionalized by setting $\tilde{p}=p/p_{e}$ where *p*_*e*_ is the effective pressure *p*_*e*_ = *p*_*v*_ − *σ*(*π*_*v*_ − *π*_*i*_), and once again rescaling the radial distance *r* with the tumor radius *R*, gives (in spherical coordinates)

(6) $$\frac{1}{{\tilde{r}}^{2}}\frac{d}{d\tilde{r}}\left({\tilde{r}}^{2}\frac{d\tilde{p}}{d\tilde{r}}\right)=-\alpha^{2}\left(1-\tilde{p}\right),$$

where $\alpha =R\sqrt{L_{p}S/(KV)}$. In general, the nondimensional parameter *α* may depend on the concentration of growth factors; for instance, experimental studies [24] have shown that an increase of VEGF induces approximately a 5-fold increase in *L*_*p*_. Here, we assume that *α* does not change locally; however, different values are chosen for normal (*α*_*h*_), tumor (*α*_*t*_), and normalized cases [10].

The pressure parameters for host (subscript *h*) and tumor (subscript *t*) tissues along with those for tumors whose vascular structure has been normalized using antiangiogenic agents are based on Table 3 of Jain et al. [10]; these are included in Table 2. While their original table has a range for the vascular pressure *p*_*v*_, we have set a base value of *p*_*v*_ = 20 mmHg for all tissues. This value is close to the mean value for the range of parameters in both normal and tumor tissues. Similarly, the ranges for *S*/*V* are replaced with values near the mean.

### 2.4. Solution Method

We assume that the tumor is embedded in normal host tissue (e.g., in an organ) and consider the following boundary conditions. We ensure spherical symmetry at the core by imposing *df*_*j*_/*dr*|_*r*=0_ = 0 and *dp*/*dr*|_*r*=0_ = 0 and enforce continuity of factor concentrations and fluxes at the tumor boundary by setting *f*_*j*_(*R* ^−^) = *f*_*j*_(*R* ^+^) and −*D*_*j*_^*t*^∂*f*_*j*_/∂*r* + *uf*_*j*_|_*r*=*R*^−^_ = −*D*_*j*_^*h*^∂*f*_*j*_/∂*r* + *uf*_*j*_|_*r*=*R*^+^_ along with continuity of IFP and IFV by setting *p*(*R* ^−^) = *p*(*R* ^+^) and *u*(*R* ^−^) = *u*(*R* ^+^), that is, −*K*_*h*_(*dp*/*dr*)|_*r*=*R*^−^_ = −*K*_*t*_(*dp*/*dr*)|_*r*=*R*+_, where typically *K*_*h*_ = *K*_*t*_ (based on Table 3 from [10]). We also require that factor concentrations reach steady state as we move away from the tumor and so impose *f*_*j*_(*r*) → *g*_*j*_^*h*^/*k*__*j*__^*h*^( = *f*_*j*_^*s*^) for large *r*. Finally, assuming that normal tissue will eventually drain all excess interstitial fluid we have *p*(*r*) → 0 for large *r* [10]. 

The analytical solution for nondimensionalized pressure, $\tilde{p}$, can be obtained by solving (6). This along with the solution for nondimensional IFV, $\tilde{u}=uR/\left(K_{t}p_{e}\right)$, was derived by Jain et al. [10] (see Supplement 1 in Supplementry Material available online at doi: 10.1155/2011/843765). The radial profile for $\tilde{p}$ is then used to numerically solve for the factor concentrations in (3). The corresponding matrix inversion problem was performed using a second-order finite difference scheme in Matlab.

## 3. Results

**Figure 1:**
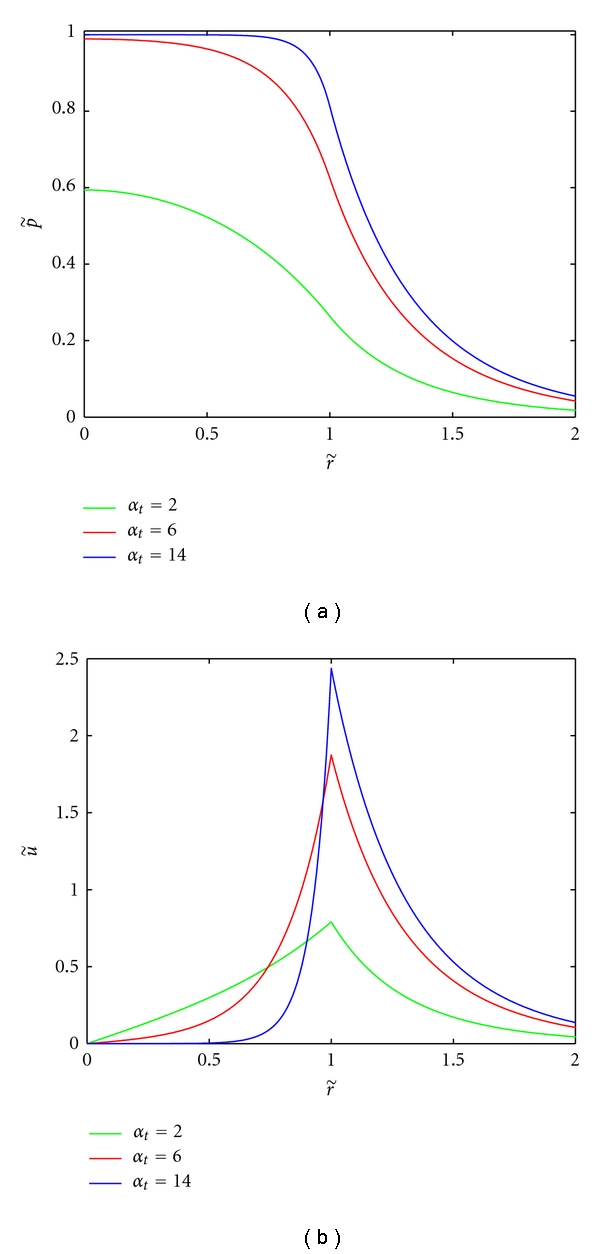
Relative pressure and velocity profiles when *α*_*h*_ = 2. Relative IFP is $\tilde{p}=p/p_{e}$ and relative IFV is $\tilde{u}=uR/\left(K_{t}p_{e}\right)$. Legend entries correspond to approximate *α*_*t*_ values for normal tissue, normalized, and nonnormalized tumor tissues, respectively. The tumor boundary is at $\tilde{r}=1$.

Interstitial pressure and velocity profiles are obtained from solving (6); these profiles can be viewed in Figures 1(a) and 1(b), respectively. As the parameter *α*_*t*_ increases, the pressure reduction at the tumor rim becomes more drastic, it also leads to higher IFP inside the tumor. Equivalently, as *α*_*t*_ increases, the interstitial fluid velocity reaches a higher and sharper peak at the tumor rim. Note that the parameter choices for *α*_*t*_ inside the tumor of 2, 6, and 14 correspond roughly to parameters associated with normal tissue, normalized tumor tissue, and nonnormalized tumor tissue, respectively; the choice for *α*_*h*_ outside the tumor remains a constant value of 2 in Figures 1(a) and 1(b) [10].

**Figure 2:**
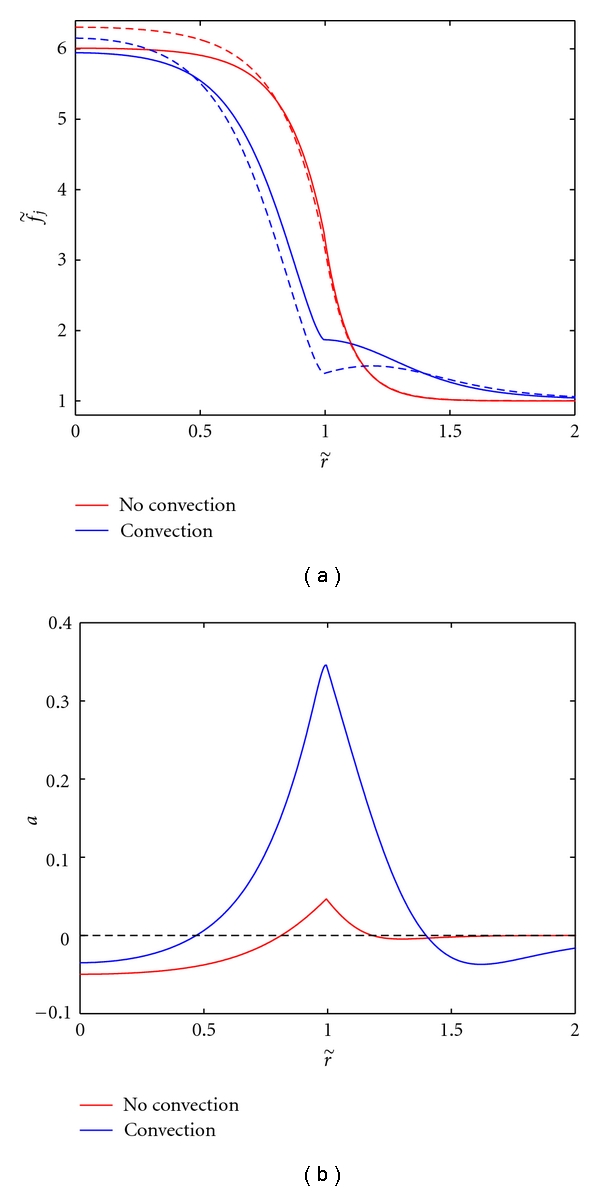
Comparing nondimensionalized proangiogenic (solid) and antiangiogenic (dashed) growth factor concentrations (Figure 2(a)) and angiogenic activity (Figure 2(b)) for the model without convection (*u* = 0) and with convection (*α*_*t*_ = 14, *α*_*h*_ = 2). With the addition of convection, the area of angiogenic stimulation is larger and more pronounced.

We solve (3), with the boundary conditions given in the previous section and the pressure profiles obtained from solving (6), to determine AGF concentrations and subsequently the imbalance factor in (4). First, we compare the results of our model with those of the model without convection [11]; the only difference between the curves corresponding to no convection in Figure 2(a) and the original results of [11] is that we consider a tumor radius of 4 mm (up from 2.5 mm) in order to remain consistent with the parameter estimates from [10] that are used for the pressure model. For our model with convection, the angiogenic activity still reaches a maximum at the tumor boundary, and the region of angiogenic suppression persists at the tumor core. However, we now observe a higher peak of angiogenic tendency at the rim and a larger area of angiogenic stimulation, see Figure 2(b). This is due to the larger difference between the proangiogenic and antiangiogenic growth factor concentrations near the boundary, a result of these factors being pushed out of the tumor core into the surrounding normal tissue, see Figure 2(a). However, as we will see, the transition from a system without convection to a system with convection is not straightforward.

By changing the values of *α*_*t*_, we can see the effect of varying only the contribution of the pressure profile on the model. This can be achieved by modifying *L*_*p*_, *S*/*V*, or *R*. However, we wish to consider a fixed tumor radius and surface to volume ratio, so we will consider changes in *α*_*t*_ to correspond to changes in the values of *L*_*p*_ only. Note that we cannot analyze the contribution of IFP alone by modifying *K* since this would also change the value of the nondimensional convection parameter in (3). Similarly, we can look at the contribution of the nondimensional convection parameter alone by modifying *p*_*e*_. Finally, we will consider the effect of changing the hydraulic conductivity *K* which simultaneously decreases *α*_*t*_ and increases ${\tilde{K}}_{j}$ affecting the pressure profile and the rate of convection.

**Figure 3:**
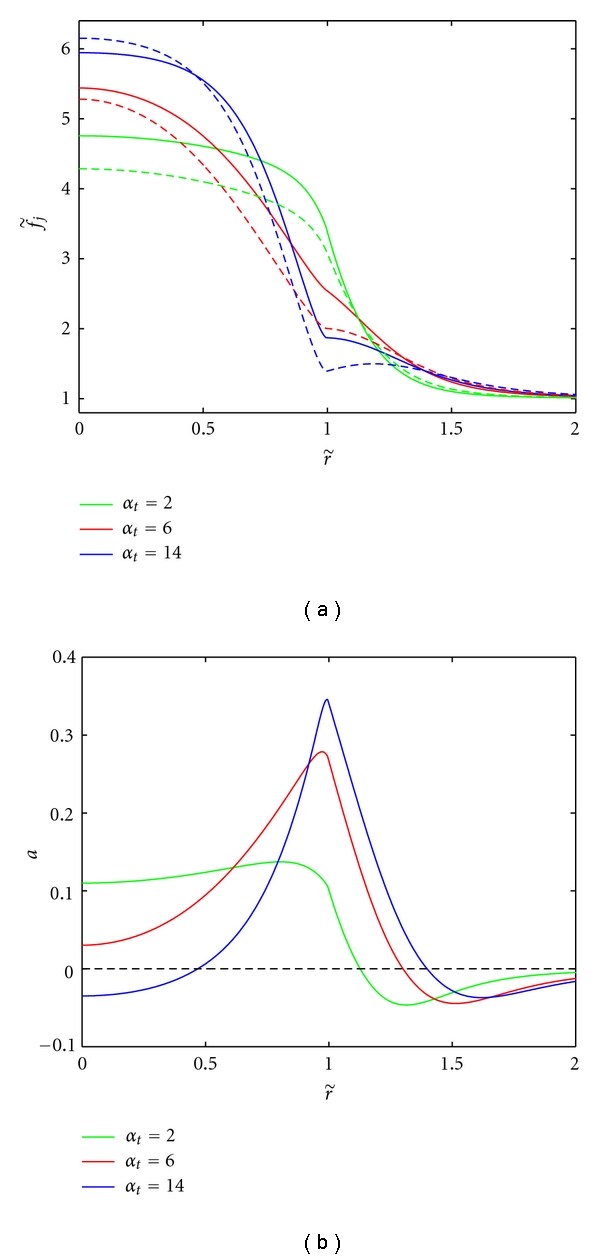
Effect of varying the vascular hydraulic permeability, *L*_*p*_ (mm/s/mm Hg), in the tumor tissue on AGF concentrations (Figure 3(a)) and angiogenic activity (Figure 3(b)). The values of *α*_*t*_ = 2, 6, 14 correspond to *L*_*p*_ = 3.6 × 10^−7^, 3.7 × 10^−6^, 1.86 × 10^−5^ mm/s/mm Hg. The parameter for host tissue was fixed at *α*_*h*_ = 2. This changes the shape of the resulting pressure profile and thus the interstitial fluid velocity.

**Figure 4:**
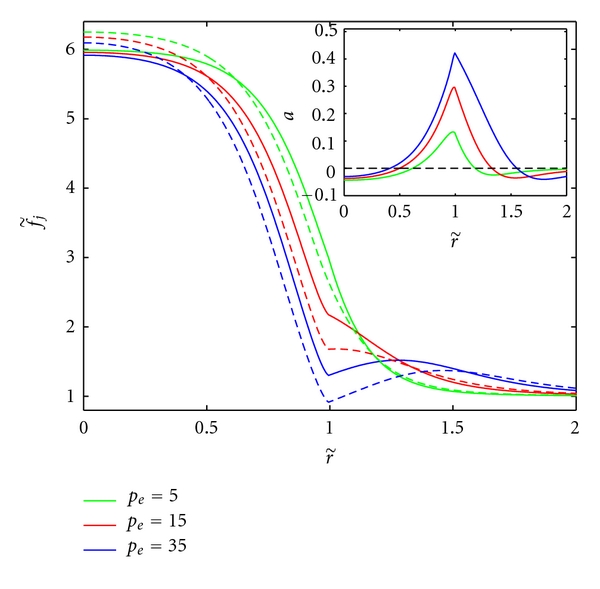
The effect of varying *p*_*e*_ (mm Hg) on AGF concentrations (inset) and angiogenic activity for fixed *L*_*p*_ = 1.86 × 10^−5^ mm/s/mm Hg. This alters the value of the convection parameters ${\tilde{K}}_{j}$.

**Figure 5:**
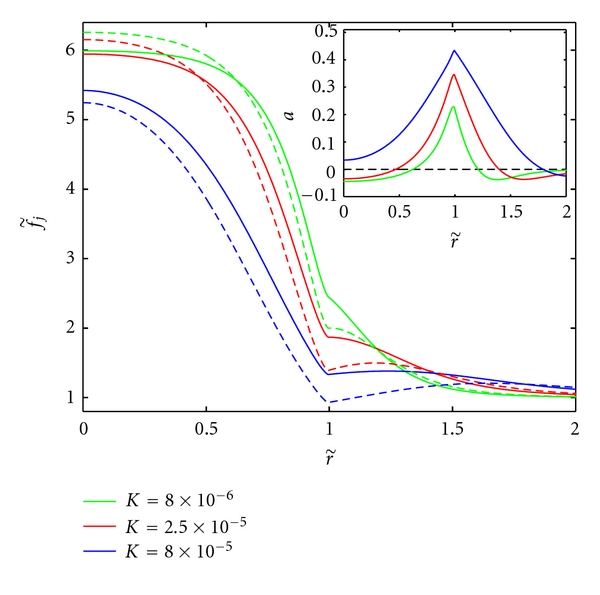
The effect of increasing *K* (mm^2^/s/mm Hg) on AGF concentrations (inset) and angiogenic activity for fixed *L*_*p*_ = 1.86 × 10^−5^ mm/s/mm Hg and *p*_*e*_ = 10.9 mm Hg. This simultaneously decreases *α*_*t*_ and increases ${\tilde{K}}_{j}$ leading to altered IFP and elevated convective transport.

We consider first the effects of varying only the pressure parameter *α*_*t*_ (for fixed *α*_*h*_ = 2), which, as stated above, we will assume is achieved by changing *L*_*p*_, leaving the rest of the parameters fixed as in Table 2. As can be seen in Figures 3(a) and 3(b), the results suggest that altering the pressure parameter leads to different angiogenic behavior in the tumor. Indeed, the region of angiogenic suppression (*a* < 0) at the core is not conserved for all values of *α*_*t*_ between the cases of negligible convection (*α*_*t*_ ≈ 0) and nonnormalized tumor tissue (*α*_*t*_ = 14). Thus, the behavior is more complicated than that observed by modifying the convection parameter via *p*_*e*_ or both pressure and convection parameters via *K* (see Figures 4 and 5 and Discussion below). For values of *α*_*t*_ close to zero, there is a region of angiogenic suppression (*a* < 0) at the core and stimulation (*a* > 0) at the rim (similar to the “no convection” case in Figure 2(b)). For midrange values of *α*_*t*_ the tumor can experience global angiogenesis (i.e., *a* > 0 for all *r* ∈ [0, *R*]). That is, instead of the proangiogenic and antiangiogenic concentrations balancing inside the tumor, the antiangiogenic factor concentration lies entirely below that of the proangiogenic factor concentration. Considering *α*_*t*_ = 2, this assumes that the tumor tissue has the same value of *L*_*p*_ as normal tissue. Indeed, the angiogenic activity in this case remains constant inside the tumor due to a consistent difference between the two factor concentrations leading to global angiogenesis. For the parameters corresponding to normalized tumor tissue (*α*_*t*_ = 6), we see an intermediate behavior where global angiogenesis occurs, but the activity at the core is much lower than at the tumor rim. For high values of *α*_*t*_ (e.g., *α*_*t*_ = 14), the core once again becomes a region of suppression but with higher levels of angiogenic activity occurring close to the tumor rim as previously observed in the “convection” case in Figure 2(b). While different angiogenic profiles are obtained, it remains true that the angiogenic activity at the tumor rim increases with *α*_*t*_.

As expected, fixing *α*_*t*_ = 14 and increasing *p*_*e*_, thereby increasing the convection of factors leads to higher concentrations outside the tumor (see Figure 4) and larger concentration differences at the rim. This coincides with an increase of angiogenic activity at the tumor rim. For the parameters considered and a reasonable range of *p*_*e*_, the tumor exhibits only focal suppression. Other activities can be achieved but require a modification of the AGF parameters (see the sensitivity analysis in Figures  S1–S3).

Finally, we consider varying *K*, an increase in this parameter value leads to decreased *α*_*t*_ and *α*_*h*_ along with increased values of the convection parameters ${\tilde{K}}_{j}$. This essentially compounds the effects observed in Figures 3(a), 3(b) and 4 leading to elevated levels of angiogenesis inside the tumor (from decreasing *α*_*t*_) and at the tumor rim (from increasing *p*_*e*_); refer to Figure 5.

Following Ramanujan et al. [11], we performed a sensitivity analysis on AGF production; see the Supplementary Figures  S1–S3. We generalize their results by fixing one of the parameters of interest (*α*_*t*_, *p*_*e*_, and *K*) and analyze results over a large range of tumor production values (${\tilde{g}}_{p}^{t}$ and ${\tilde{g}}_{a}^{t}$ **)**. Focal suppression occurs between lines of the same color, global suppression occurs above this region (high ${\tilde{g}}_{a}^{t}$ low ${\tilde{g}}_{p}^{t}$), and global angiogenesis occurs above (low ${\tilde{g}}_{a}^{t}$, high$\;{\tilde{g}}_{p}^{t}$). The results indicate that focal suppression is observed only for a narrow sliver of the parameter space. This is realistic since one would assume that the behavior is sensitive to the balance of these factors' production rates. As expected, the behavior is not as sensitive to varying the host production parameters as it is to tumor production (results are not shown).

Figure  S1 shows how this sensitivity changes when varying *α*_*t*_. As *α*_*t*_ increases, the region of focal suppression widens from a very narrow region to encompass more of the parameter space above and below this region. For different values of *p*_*e*_, we notice little movement in the boundary between focal suppression and global angiogenesis while the boundary between focal suppression and global suppression increases with *p*_*e*_ (see Figure  S2). Finally, for *K*, we see a combination of these effects; the region where focal suppression and peripheral stimulation occur is widening and drifting towards the top left corner of Figure  S3 (high$\;{\tilde{g}}_{a}^{t}$, low ${\tilde{g}}_{p}^{t}$). Parameter values that correspond to focal suppression switch to values corresponding to globally angiogenic behavior when the convection term is added. Also shown in these graphs are the precise points in parameter space corresponding to the tumor production values ${\tilde{g}}_{p}^{t}=349$ and ${\tilde{g}}_{p}^{t}=279$ (see Section 4).

## 4. Discussion

We have presented a mathematical model to study the effects of interstitial convection on proangiogenic and antiangiogenic factor concentrations in tumor and surrounding host tissue, and from this determined the overall angiogenic activity of the tumor. The resulting AGF concentration profiles agree qualitatively with experimental observations that show the highest concentrations in the core of the tumor, decreasing as one approaches the tumor rim [4]. Also, the resulting angiogenic behaviors, including suppression at the tumor core and maximal angiogenic stimulation near the tumor rim, correspond with experimental observations such as tumor perfusion [25]. The imbalance between proangiogenic and antiangiogenic factors provides an empirical explanation for observed angiogenic activity and could be correlated with resulting tumor necrosis or growth. While the precise effects of IFP and factor convection on the angiogenic activity of tumors have not been experimentally verified, our results indicate that an IFP gradient could significantly influence suppression and stimulation of angiogenesis in a tumor.

While not explicitly included, the effect of antiangiogenic treatments can be ascertained in this model since it has been shown that the value of *α*_*t*_ decreases when the vasculature is normalized due to the application of, for instance, anti-VEGFR-2 [10]. The value of *α*_*t*_ can decrease through any combination of decreasing *S*/*V*, *L*_*p*_, or *R*, all of which could occur as a result of antiangiogenic therapy. *S*/*V* decreases as blood vessels are destroyed and/or remodeled due to the administration of an antiangiogenic drug (in this case, there is less area of vessel wall per unit of tumor volume). The radius *R* decreases indirectly; as tumor vessels regress, tumor cells are deprived of oxygen and consequently become hypoxic or necrotic. Moreover, the vessel permeability *L*_*p*_ could decrease due to judicious application of antiangiogenic agents (vessel normalization, [2]). The pressure parameter *α*_*t*_ could also be decreased by increasing the hydraulic conductivity *K*, an effect that would be somewhat counterbalanced by the corresponding increase in the convection parameter (refer to Figure  5). However, *K* could be increased by administering enzymes that degrade the extracellular matrix (ECM), which subsequently decreases the flow resistance in the interstitium [21]. Overall, strategies that lead to the reduction of the parameter *α*_*t*_ result in decreased IFP, which influences tumor angiogenesis activity. As shown in Figure 3(b) and discussed in Section 3, the changes in the interstitlal fluid velocity from decreasing *α*_*t*_ reduces the angiogenic activity at the rim and produces a more constant level of angiogenic activity inside the tumor. On the other hand, one could also consider the administration of antiangiogenic agents by decreasing the concentration of proangiogenic factors through an increase of their degradation (or deactivation) constant (see Figure  S5). This increase in deactivation should take into account that most antiangiogenic treatments only affect a single factor or a family of growth factors.

One can consider the effect of cytotoxic therapies by noting that the application of either chemotherapy or radiotherapy reduces the number of tumor cells leading to less proangiogenic factor production (Figure  S4); this is achieved by lowering the parameter ${\tilde{g}}_{p}^{t}$. We considered the reduction of this production rate and the movement through parameter space is shown in the sensitivity diagrams (Figures  S1–S3). In Figure  S4, we can observe that the resulting angiogenic behavior depends on the value of *α*_*t*_ as summarized in Table 3. The effect of cytotoxic therapies either on tumor cells or blood vessels could also reduce the pressure due to increased interstitial space; this could be included in our model by, for instance, increasing *K* (Figure 5). The effects of cytotoxic treatments could also be included by reducing the tumor radius *R*. Combinations of antiangiogenic therapy and chemotherapy could be considered by performing the aforementioned parameter changes simultaneously; these changes compound the effects leading to even further reduced angiogenic tendency.

Decreasing the IFP prior to or simultaneously with other therapies is an important concern in cancer treatment since the flow of interstitial fluid out of the tumor prevents drugs from penetrating the tumor bulk. While the various effects of antiangiogenic treatments (decreasing *S*/*V*, *L*_*p*_, or *R*) or ECM-degrading enzymes (increasing *K*) all reduce *α*_*t*_ and hence pressure, there are other independent mechanisms that could also elicit reductions in pressure. For instance, reducing *p*_*e*_ would reduce IFP. This could be achieved by reducing the vascular pressure *p*_*v*_, which can be readily accomplished by decreasing the resistance of blood. Clinically, decreasing the viscosity of the blood or normalizing the tumor vasculature would accomplish the goal of less resistance [21]. The effects of changing *p*_*e*_ are shown in Figure 4 as decreasing the effective pressure leads to diminishing levels of angiogenic activity at the rim.

By taking into consideration the different angiogenic behaviors exhibited by modifying any of the key parameters involved in the pressure model, we can establish an alternate (or more likely, complementary) mechanism for these changes. Whereas it was previously hypothesized [11] that the production, degradation, and diffusion of the AGFs were primarily responsible for the overall angiogenic behavior, we have exhibited that changes in the tumor tissue physiology could also elicit these changes. The interplay between the two groups of parameters, those related to AGF properties that determine AGF concentrations and those corresponding to the tumor physiology that determine interstitial fluid pressure, should be further investigated with more detailed modeling and experimental work. We emphasize that it was never the goal of this work to quantitatively predict concentrations of specific AGFs or to model the process of angiogenesis but rather to emphasize the importance of tumor tissue properties and macromolecule convection on angiogenic behavior.

One should note that there are limitations to our mathematical model, many of which have been mentioned during the model development. These include the existence of two distinct groups of AGFs, the specific functional form of our angiogenic activity measure and the distributed fluid source terms. Most prominent among these various assumptions are those of spherical symmetry and homogeneity of tissues and environment [11]. The other main limitation is the assumption of constant parameter values both inside the tumor and in the host tissue, or even the constant values assumed across this boundary. These assumptions make the modeling and computation tractable especially since physiological parameters as functions of radial distance are uncommon in the literature. However, our computational approach can readily be extended to include aspects of the heterogeneous tumor microenvironment upon availability of relevant experimental data. Finally, we propose that an experimental study measuring both interstitial fluid pressure and quantities associated with AGF concentrations or angiogenic activity (such as vessel density or perfusion) would help to validate our qualitative model predictions.

## Figures and Tables

**Table 1:** Molecular weights of common proangiogenic and antiangiogenic growth factors.

Molecule	Angiogenic category	Size (kDa)
VEGF_165_ dimer	Proangiogenic	45 [13]
FGF family	Proangiogenic	17–34 [14]
TSP-1	Antiangiogenic	140 [15]
Angiostatin	Antiangiogenic	38 [16]
Endostatin	Antiangiogenic	20 [17]

**Table 2:** Model parameters [10, 11].

Parameter	Units	Host	Tumor	Normalized
*R*	mm	—	4	4
*K*	mm^2^/s/mm Hg	2.5 × 10^−5^	2.5 × 10^−5^	2.5 × 10^−5^

Angiogenic growth factors				

*D_p_*	mm^2^/s	4.0 × 10^−5^	5.5 × 10^−5^	5.5 × 10^−5^
*D_a_*	mm^2^/s	3.25 × 10^−5^	4.0 × 10^−5^	4.0 × 10^−5^
*k_p_*	s^−1^	2.0 × 10^−4^	1.99 × 10^−4^	1.99 × 10^−4^
*k_a_*	s^−1^	1.5 × 10^−4^	1.1 × 10^−4^	1.1 × 10^−4^
*g*_*p*_	*μ*g/mm^3^/s	2.0 × 10^−4^	12.0 × 10^−4^	12.0 × 10^−4^
*g*_*a*_	*μ*g/mm^3^/s	1.5 × 10^−4^	7.0 × 10^−4^	7.0 × 10^−4^
${\tilde{k}}_{p}$	—	80	57.9	57.9
${\tilde{k}}_{a}$	—	73.8	44	44
${\tilde{g}}_{p}$	—	80	349	349
${\tilde{g}}_{a}$	—	73.8	280	280
${\tilde{K}}_{p}$	—	12.5	9.1	9.1
${\tilde{K}}_{a}$	—	15.4	12.5	12.5

Interstitial fluid pressure				

*L*_*p*_	mm/s/mm Hg	3.6 × 10^−7^	1.86 × 10^−5^	3.7 × 10^−6^
*S*/*V *	mm^2^/mm^3^	17.4	16.5	15.2
*p*_*v*_	mm Hg	20	20	20
*σ*	—	0.91	8.7 × 10^−5^	2.1 × 10^−3^
**π*_v_*	mm Hg	20	19.8	19.2
**π*_i_*	mm Hg	10	17.3	15.1
*p*_*e*_	mm Hg	10.9	20	20
*α*	—	2	14	6

**Table 3:** Angiogenic activity resulting from cytotoxic therapy (see Figure S4).

*α*_*t*_	${\tilde{g}}_{p}^{t}=349$ (no treatment)	${\tilde{g}}_{p}^{t}=279$ (cytotoxic therapy)
2	Global angiogenesis	Global suppression
6	Global angiogenesis	Focal suppression
14	Focal suppression	Focal suppression
